# 2276. Utilization of Vancomycin in the Emergency Department at a Community Hospital and Implications of its Use

**DOI:** 10.1093/ofid/ofad500.1898

**Published:** 2023-11-27

**Authors:** Danielle Snodgrass, Kristina E White, Aline Azar, Praveen Sudhindra

**Affiliations:** Carle Health, Peoria, Illinois; Carle Health, Peoria, Illinois; Carle Health, Peoria, Illinois; Carle Health, Peoria, Illinois

## Abstract

**Background:**

Antimicrobial Stewardship in the Emergency Department has many challenges. Our region performed a utilization review of vancomycin prescribed in the Emergency Department over a 2-month period in 2022.

In this utilization review, we sought to examine vancomycin continuation when a patient was admitted from the Emergency Department, whether vancomycin was indicated for the suspected diagnosis, and if a potential vancomycin-induced adverse drug event (ADE) occurred from its administration.

**Methods:**

A retrospective chart review across three campuses of a hospital system examined 265 emergency room encounters where vancomycin was ordered and administered. Data was recorded on concurrent antibiotic administration, the order set utilized and indication when ordering, admission versus discharge of the patient, continuation of vancomycin upon admission, and appropriateness of vancomycin for the documented indication. Adverse drug event data (Table 2) was recorded, when possible, within seven days of vancomycin administration. This included the presence of acute kidney injury, thrombocytopenia, neutropenia, or a possible drug-induced rash.

**Results:**

Results are summarized in Tables 1-3.

**Figure:**
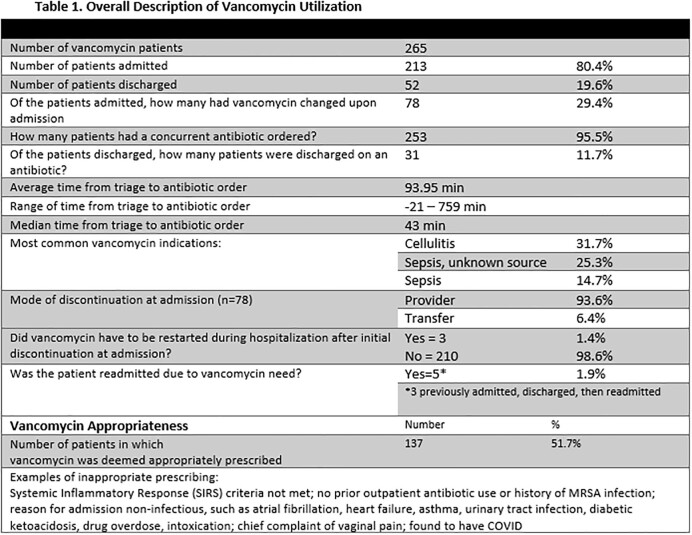

**Figure:**
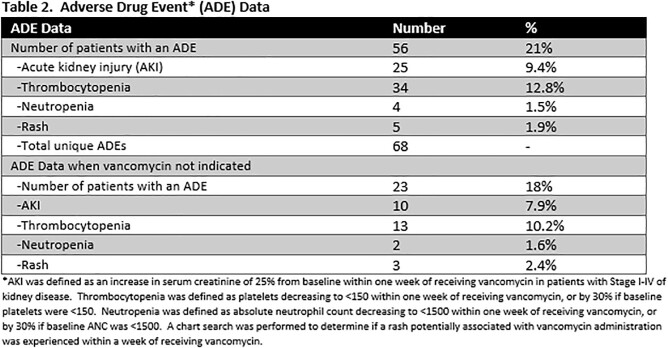

**Figure:**
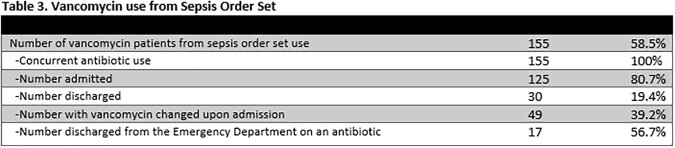

**Conclusion:**

Our findings showed that there are negative implications associated with vancomycin use from the Emergency Department. Data revealed that only 51.7% of patients who received vancomycin truly had indications for its use based on the suspected diagnosis, and 29.4% of patients who were admitted subsequently had vancomycin discontinued upon admission. In addition, 19.6% of patients were discharged directly from the Emergency Department, never being admitted.

More importantly, 21% of patients experienced an ADE following vancomycin administration. With half of vancomycin use being categorized as unnecessary, this number emphasizes that harm can, and often does, occur with unnecessary vancomycin administration. The most common ADEs were thrombocytopenia at 12.8% and acute kidney injury at 9.4%. This review brings further attention and awareness to the need for Antimicrobial Stewardship in the Emergency Department. More guidance should be offered for triaging appropriate empiric antibiotics when infection is present.

**Disclosures:**

**All Authors**: No reported disclosures

